# A Preventable Adverse Reaction: Drug Reaction With Eosinophilia and Systemic Symptoms (DRESS) Syndrome Following Allopurinol Use for Asymptomatic Hyperuricemia

**DOI:** 10.7759/cureus.91354

**Published:** 2025-08-31

**Authors:** Hadiza Ibrahim, Ghada Rashwan, Adil Jumani, Hana Purra, Alhasssan Alteneiji, Syed Hussaini

**Affiliations:** 1 Internal Medicine, Zayed Military Hospital, Abu Dhabi, ARE

**Keywords:** allopurinol hypersensitivity, asymptomatic hyperuricemia, drug-induced rash, drug reaction, drug reaction with eosinophilia and systemic symptoms

## Abstract

Drug reaction with eosinophilia and systemic symptoms (DRESS) is a potentially life-threatening drug-induced hypersensitivity reaction. We report the case of a 36-year-old man who developed DRESS syndrome after starting allopurinol for presumed asymptomatic hyperuricemia. He presented with fever and a widespread rash. Symptoms developed within 10 days of starting allopurinol. Labs showed transaminitis, delayed-onset eosinophilia, and elevated inflammatory markers. Biopsy confirmed a drug-induced hypersensitivity pattern. Of note, serum uric acid was normal on admission to our facility. The patient improved with corticosteroids. This case highlights the need for cautious prescribing and recognition of evolving features of DRESS.

## Introduction

Drug reaction with eosinophilia and systemic symptoms (DRESS) is a rare but potentially life-threatening drug-induced hypersensitivity reaction. It typically presents with fever, widespread skin eruptions, eosinophilia, and internal organ involvement, most commonly affecting the liver, kidneys, or lungs [1-3]. Onset is usually delayed, occurring two to six weeks after exposure to the inciting agent, and clinical features may evolve, making early diagnosis challenging [2].

Allopurinol is among the most frequently implicated drugs in DRESS, particularly in patients with renal impairment or those carrying the HLA-B*58:01 allele [4,5]. Despite these known risks, allopurinol continues to be prescribed inappropriately for asymptomatic hyperuricemia, a practice discouraged by recent international guidelines [6]. Asymptomatic hyperuricemia refers to elevated serum uric acid in the absence of clinical manifestations such as gout, nephrolithiasis, or urate nephropathy [6].

Recognition of early cutaneous and systemic signs, along with awareness of high-risk medications, is essential to reducing morbidity and preventing progression. Although the precise pathophysiology of DRESS is incompletely understood, it is thought to involve drug-specific T-cell activation and immune dysregulation, with frequent viral reactivation (especially human herpesvirus 6 (HHV-6), Epstein-Barr virus (EBV), and cytomegalovirus (CMV)) contributing to disease severity and relapses [2,7,8]. Management typically involves the prompt withdrawal of the offending agent and systemic corticosteroids; however, other immunomodulatory strategies, including cyclosporine and emerging biologic therapies such as IL-5/IL-5 receptor antagonists, have also been reported in severe cases [9,10].

Here, we describe a case of allopurinol-induced DRESS in a previously well adult, highlighting diagnostic considerations, biopsy findings, and therapeutic response.

## Case presentation

A 36-year-old man with type 2 diabetes mellitus was reportedly started on a daily dose of allopurinol at an outside facility for asymptomatic hyperuricemia. The patient could not recall the dose, and laboratory records from that encounter were unavailable. On admission to our hospital, his serum uric acid level was 200 μmol/L, within the normal range. He had no prior history of gout, nephrolithiasis, or uric acid nephropathy.

**Figure 1:**
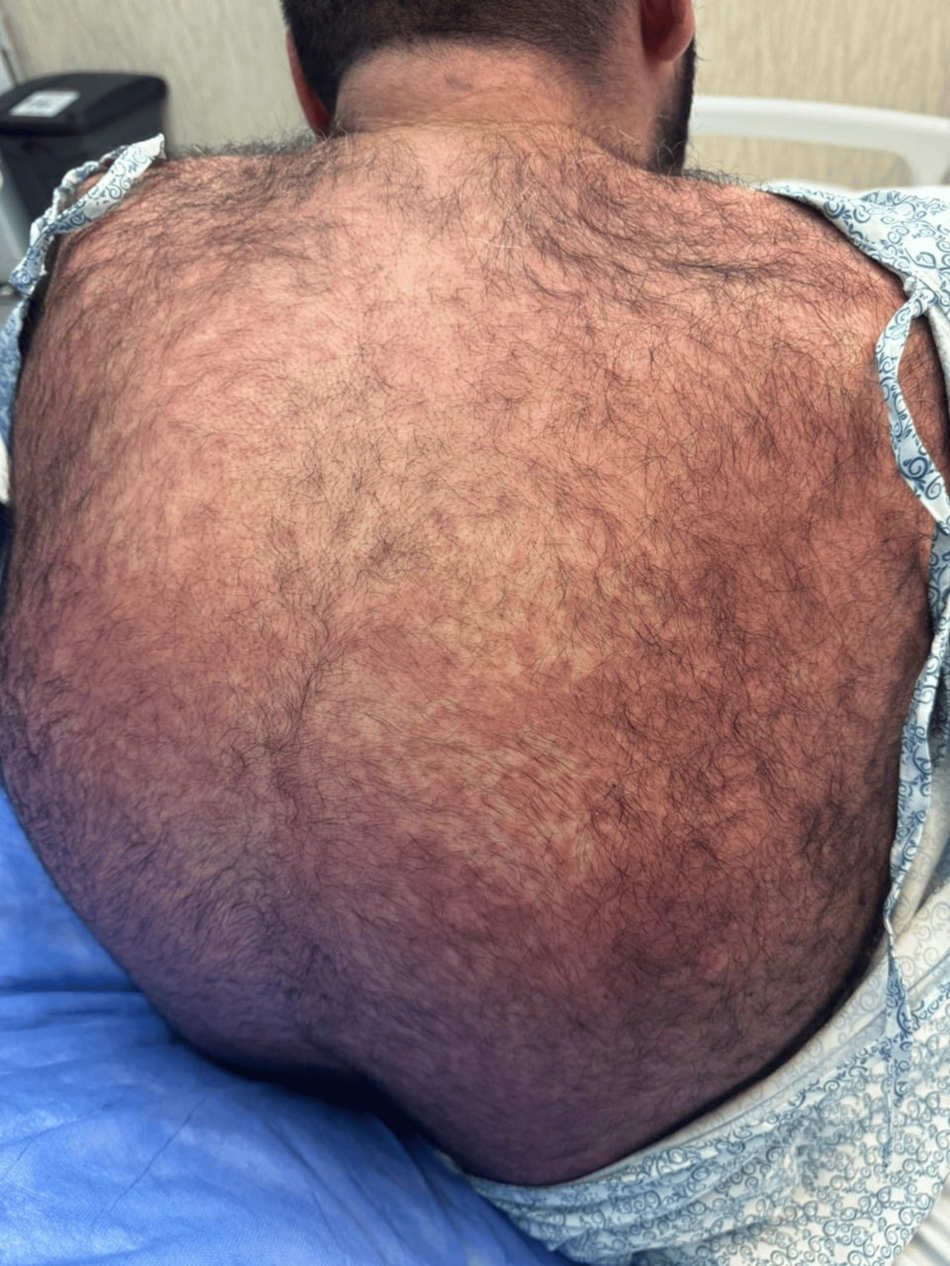
Upper back: diffuse erythematous eruption over the upper back with mild infiltration and fine scaling, consistent with the widespread morbilliform rash typical of drug reaction with eosinophilia and systemic symptoms (DRESS) syndrome.

**Figure 2:**
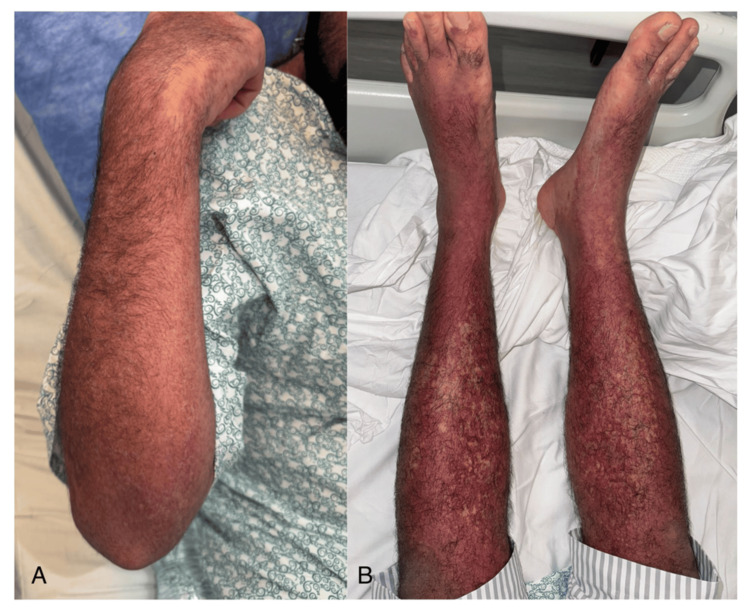
Cutaneous manifestations of drug reaction with eosinophilia and systemic symptoms (DRESS) syndrome (A) Right forearm showing erythematous and violaceous discoloration with subtle infiltration and absence of blistering or sloughing, representative of the widespread cutaneous involvement seen in DRESS syndrome. (B) Legs showing diffuse violaceous erythema with patchy distribution and subtle overlying scaling.

**Figure 3:**
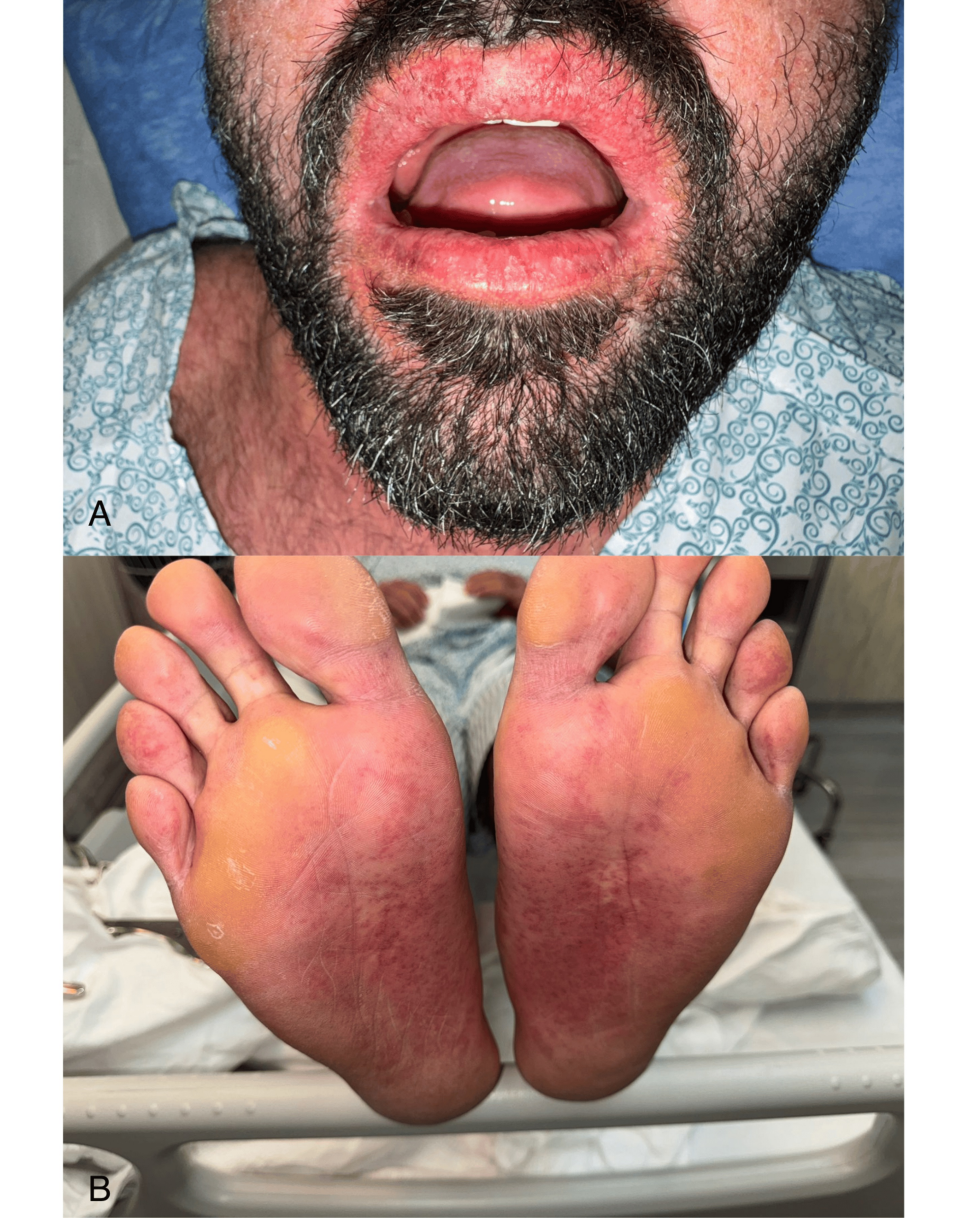
Mucocutaneous involvement in drug reaction with eosinophilia and systemic symptoms (DRESS) syndrome (A) Lips showing diffuse erythema and superficial fissuring with loss of the normal vermilion border. (B) Soles of the feet with erythematous and violaceous discoloration, scattered petechiae, and no evidence of blistering or desquamation.

**Figure 4:**
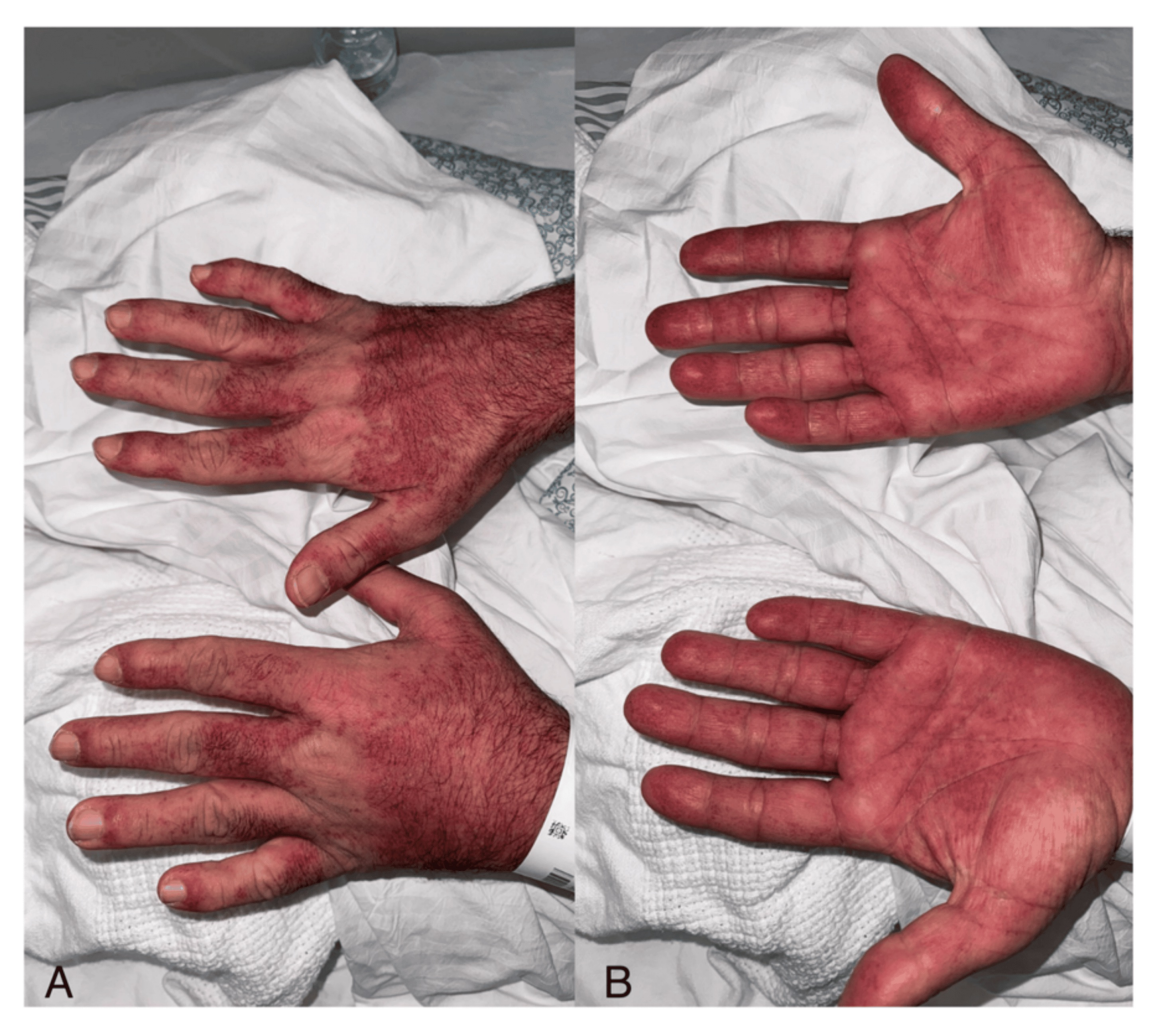
Hand involvement in drug reaction with eosinophilia and systemic symptoms (DRESS) syndrome (A) Dorsum of the hands showing violaceous erythema with subtle infiltration and no blistering or necrosis. (B) Palmar surfaces demonstrating diffuse erythema with patchy violaceous discoloration, without peeling or sloughing.

Ten days after starting allopurinol, he presented with high-grade fever (up to 39.2°C), malaise, and a morbilliform rash that began on the trunk and spread to the limbs, palms, soles, and lips. There was no ocular or genital involvement (Figures 1-4). On examination, he appeared unwell with a heart rate of 118 bpm, blood pressure of 119/75 mmHg, and SpO₂ of 98% on room air.

Initial labs revealed leukocytosis (14.5 ×10⁹/L) with normal eosinophil count (0.1 ×10⁹/L), alanine aminotransferase (ALT) 210 U/L, aspartate aminotransferase (AST) 185 U/L, alkaline phosphatase (ALP) 350 U/L, C-reactive protein (CRP) 61 mg/L, and procalcitonin 2.93 ng/mL. Renal function was normal. Viral serologies (hepatitis B virus (HBV), hepatitis C virus (HCV), HIV, CMV, EBV) and cultures were negative. Imaging was unremarkable. A summary of the laboratory findings is presented in Table 1.

**Table 1:** Summary of laboratory results on admission with peak eosinophil count during hospitalization ALP: alkaline phosphatase, ALT: alanine aminotransferase, AST: aspartate aminotransferase, CRP: C-reactive protein

Parameter	Result	Reference Range
White Blood Cells (×10⁹/L)	14.5	4.0 – 11.0
Eosinophils (×10⁹/L)	0.1 → 0.97	0.04 – 0.4
ALT (U/L)	210	7 – 56
AST (U/L)	185	5 – 40
ALP (U/L)	350	44 – 147
CRP (mg/L)	61	<5
Procalcitonin (ng/mL)	2.93	<0.5
Serum Creatinine (mg/dL)	Normal	0.6 – 1.2
Viral Serologies	Negative	—
Blood & Urine Cultures	Negative	—

**Figure 5:**
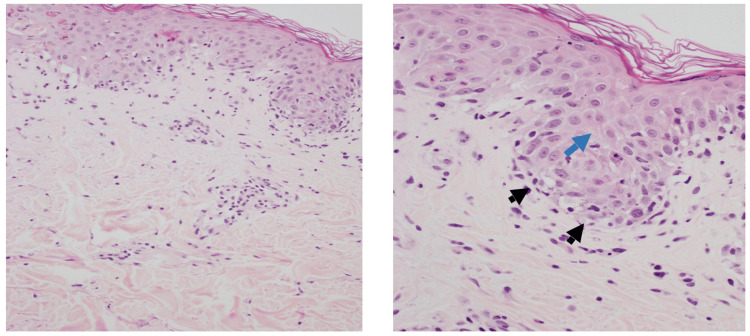
Skin (left image) shows normal basket woven keratin, and mild dermal inflammatory cells. At higher magnification, the right image shows epidermal spongiosis (spaces between keratinocytes - blue arrow) and vacuolar interface dermatitis (vacuolar alteration at the demo-epidermal junction- black arrow head).

**Figure 6:**
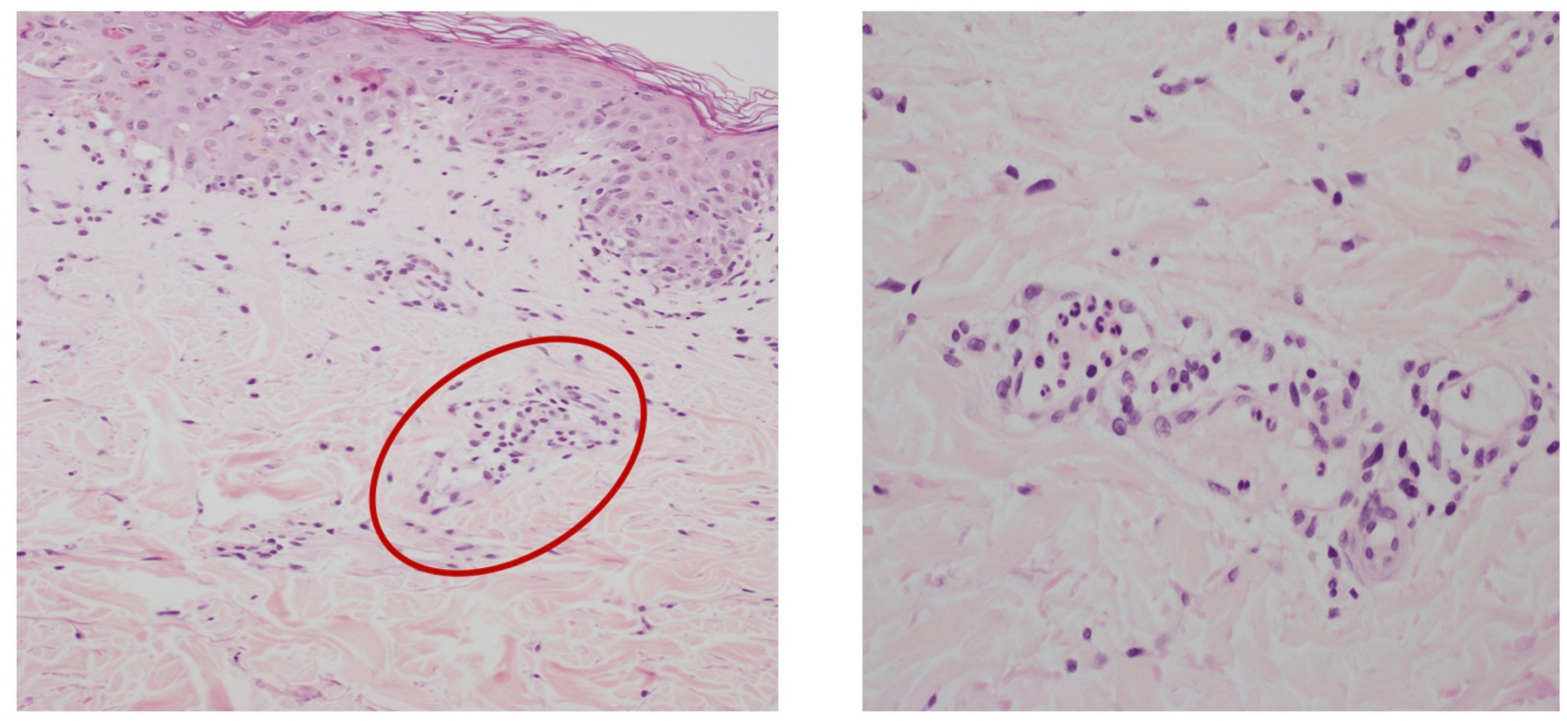
The dermis shows mild perivascular inflammatory cells infiltrate with small clusters of eosinophils and rare neutrophils within vascular spaces (the left image red circle magnified in the left image).

**Figure 7:**
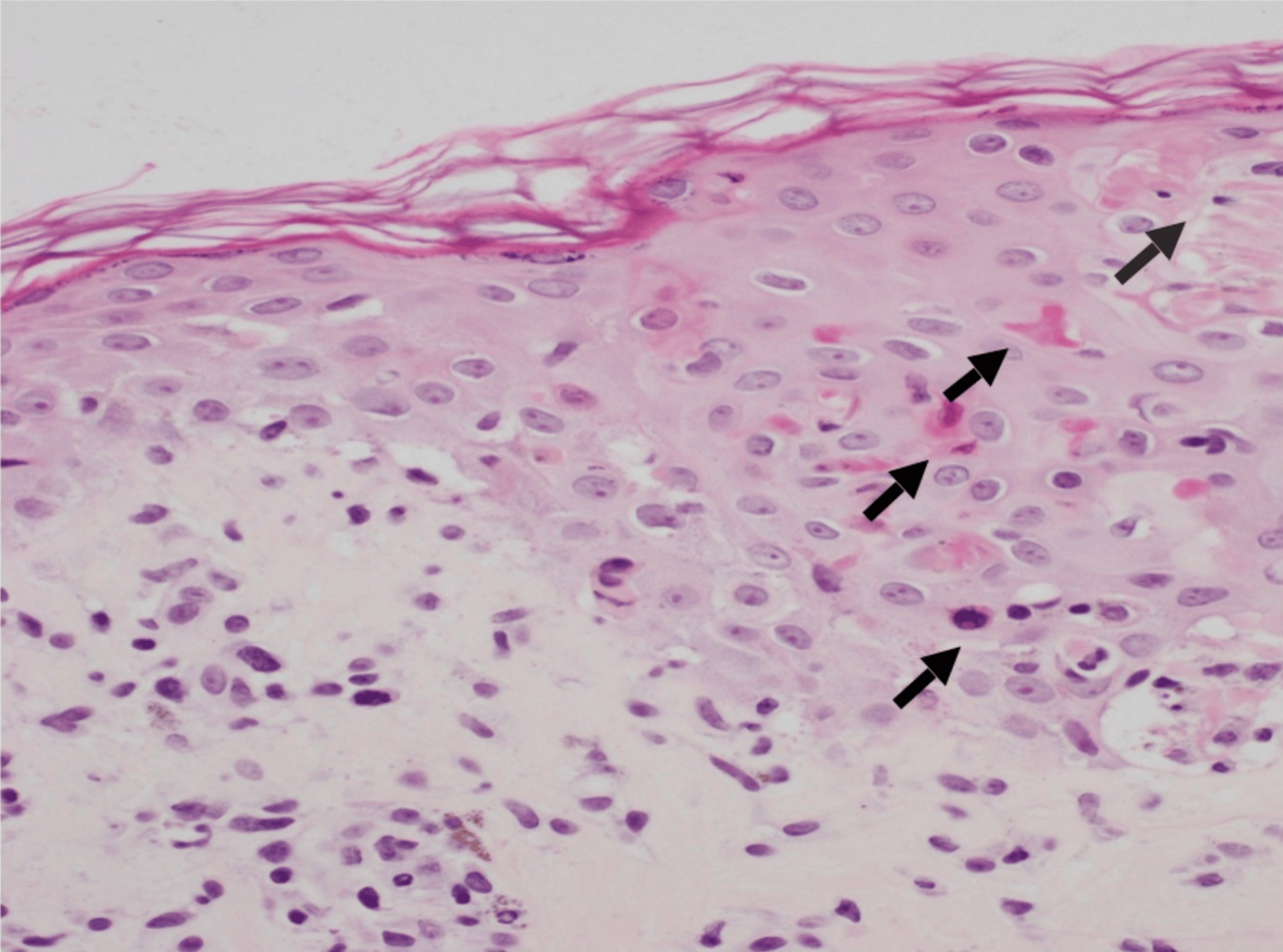
Necrotic keratinocytes at all levels of the epidermis (arrows).

Despite initiating corticosteroids at admission, his eosinophil count rose and peaked at 0.97 ×10⁹/L by day two, after an initially normal count on admission (0.1 ×10⁹/L). Skin biopsy from the arm revealed vacuolar interface dermatitis with epidermal spongiosis, necrotic keratinocytes, and a dermal perivascular infiltrate rich in eosinophils and rare neutrophils (Figures 5-7). These findings were consistent with a drug-induced hypersensitivity reaction.

Allopurinol was discontinued. He was started on intravenous methylprednisolone (1 mg/kg/day) and supportive care, and later transitioned to oral prednisone. Empiric antibiotics were initially started and discontinued after ruling out infection. The fever resolved within days, the rash desquamated gradually, and liver enzymes normalized. This highlights the rapid resolution of fever with corticosteroid initiation, while cutaneous and biochemical recovery occurred more gradually over several days. He was discharged on tapering oral prednisone and remained asymptomatic on follow-up. Follow-up laboratory studies confirmed normalization of eosinophil count and liver enzymes, consistent with sustained recovery.

## Discussion

DRESS syndrome is a severe, potentially life-threatening hypersensitivity reaction characterized by fever, widespread rash, hematologic abnormalities (most often eosinophilia), and internal organ involvement. It typically occurs two to six weeks after initiation of the offending drug, with features that may evolve over time [1,2].

In this case, the patient presented with fever, malaise, and a rapidly spreading morbilliform rash approximately 10 days after starting allopurinol. This represents an earlier onset than the typical two to six week latency described for DRESS [1,2]. The distribution included the trunk, limbs, palms, soles, and lips. Although his eosinophil count was normal on admission, it rose significantly during hospitalization, peaking on day two. This delayed eosinophilia is characteristic of DRESS and highlights the importance of serial lab monitoring when the diagnosis is suspected [2].

The skin biopsy revealed interface dermatitis with necrotic keratinocytes and a dermal eosinophilic infiltrate, consistent with drug-induced hypersensitivity. These features helped distinguish the case from other serious cutaneous adverse reactions, such as Stevens-Johnson syndrome or toxic epidermal necrolysis, where keratinocyte necrosis is usually more widespread and inflammation is less pronounced [3].

The patient’s clinical and laboratory findings yielded a RegiSCAR score of 7, fulfilling the criteria for a “definite” diagnosis of DRESS. This score included fever above 38.5°C, rash involving more than 50% of body surface area, eosinophilia greater than 0.7×10⁹/L, liver involvement, compatible histopathology, prolonged recovery, and exclusion of other causes [5].

Comparable cases of allopurinol-induced DRESS in patients without gout have been reported, many showing similar latency, hepatic involvement, and preventable circumstances [10-12]. Our case reinforces these patterns and emphasizes the avoidable nature of such adverse reactions when allopurinol is prescribed without indication. There was no clear indication for urate-lowering therapy in this patient. He had no history of gout, nephrolithiasis, or uric acid nephropathy, and his serum uric acid level was normal at presentation. Allopurinol had been started for asymptomatic hyperuricemia, a practice that is not recommended. The 2020 ACR guidelines advise against initiating allopurinol in such cases due to the lack of clinical benefit and the risk of serious adverse effects [6].

Allopurinol is one of the most commonly implicated drugs in DRESS syndrome [9], and its risk is significantly higher in individuals with renal impairment or those who carry the HLA-B*58:01 allele [4,5]. Although human leukocyte antigen (HLA) testing was not performed in this patient, it is increasingly recognized as a valuable tool, particularly in high-risk ethnic groups, and could potentially prevent avoidable harm.

Management of DRESS begins with prompt withdrawal of the offending agent and supportive care. In patients with organ involvement, systemic corticosteroids remain the mainstay of treatment. This patient responded well to intravenous methylprednisolone followed by a tapering course of oral prednisone, with gradual resolution of fever, rash, and liver enzyme abnormalities.

Similar cases of allopurinol-induced DRESS in patients without gout have been reported, many of which demonstrated hepatic involvement and variable latency [9,11]. Our findings align with these reports and reinforce the importance of avoiding allopurinol in asymptomatic hyperuricemia.

In more severe or refractory cases, additional immunomodulatory treatments such as cyclosporine, intravenous immunoglobulin (IVIG), or biologic agents targeting the IL-5 pathway (e.g., mepolizumab) may be considered [10]. However, the evidence base for these therapies remains limited, and their use is typically reserved for complicated presentations [10]. Epidemiologic data suggest that DRESS occurs in approximately one to two cases per 100,000 drug exposures, with mortality rates ranging from 3.8-10% overall and up to 25% in severe cases associated with allopurinol [9,11,12]. Viral reactivation, particularly of HHV-6, EBV, or CMV, has been reported in DRESS and may contribute to a more prolonged or relapsing course [2,13].

This case highlights several key clinical lessons. First, the features of DRESS may emerge gradually and evolve throughout admission, necessitating a high index of suspicion. Second, histopathology can support early diagnosis and guide management. Third, clinicians must exercise caution when prescribing medications like allopurinol, especially in the absence of a clear indication. Where available, HLA-B*58:01 testing may further reduce the risk of severe adverse reactions. Early recognition and timely withdrawal of the culprit drug remain the cornerstones of effective management.

## Conclusions

DRESS syndrome should be considered in patients presenting with fever, rash, eosinophilia, and systemic involvement after recent drug exposure. Histologic confirmation can guide diagnosis and avoid unnecessary treatments. Clinicians must avoid prescribing urate-lowering therapy in the absence of clear indications such as symptomatic gout or uric acid nephropathy. Where feasible, HLA-B*58:01 testing should be considered before prescribing allopurinol in high-risk ethnic groups to reduce the risk of severe hypersensitivity reactions.
